# Diagnostic Value of Protein C Depletion in Pathologies Associated with the Activation of the Blood Coagulation System

**DOI:** 10.3390/ijms26136122

**Published:** 2025-06-25

**Authors:** Daria S. Korolova, Tetyana M. Platonova, Olga V. Gornytska, Volodymyr Chernyshenko, Olexandr Korchynskyi, Serhiy V. Komisarenko

**Affiliations:** 1 Protein Structure and Functions Department, Palladin Institute of Biochemistry of NAS of Ukraine, 01054 Kyiv, Ukraine; platonovatn@gmail.com (T.M.P.); olgagorn67@gmail.com (O.V.G.); bio.cherv@biochem.kiev.ua (V.C.); 2 Molecular Immunology Department, Palladin Institute of Biochemistry of NAS of Ukraine, 01054 Kyiv, Ukraine; svk@biochem.kiev.ua; 3 Laboratory of Experimental Biology and Biochemistry & General Chemistry Department, Faculty of Medicine, University of Rzeszow, 35-959 Rzeszow, Poland

**Keywords:** protein C, blood coagulation system, thrombosis, clinical diagnostics, chronic inflammatory diseases

## Abstract

Protein C (PC) is the main anticoagulant protein of the hemostasis system. It can inhibit the blood clotting cascade before the formation of a thrombus, while its concentration can decrease significantly during strong activation of blood clotting. The PC concentration was found to decrease during systemic lupus erythematosus (SLE) (with a median of 75%) and depended heavily on the inflammation index. It was also associated with the accumulation of soluble fibrin monomeric (SFMCs) (with a median of 7 µg/mL). A low PC level was detected during severe ischemic heart disease (IHD) (with medians of 60 and 63%, respectively). These pathologies also were associated with clotting activation. During abdominal aortic aneurysm (AAA), the PC level in blood plasma before surgery was found to range from 40% to 119%. A decrease in the PC level in the blood plasma of patients with AAA before surgery, lower than 78%, was associated with high blood loss (more than 1.5 L). A decrease in the PC level can lead to an imbalance between coagulation and anticoagulation. Thus, during the treatment of complex pathologies associated with the activation of coagulation, specific attention should be paid not only to classic markers of thrombus formation but also to the state of the anticoagulant link.

## 1. Introduction

The balance within the hemostasis system plays a crucial role in the pathogenesis of conditions related to thrombosis, embolism, and bleeding [1,2,3]. The disruption of this dynamic equilibrium leads to pronounced activation of the coagulation cascade, resulting in excessive thrombin formation, the accumulation of thrombophilia markers such as soluble fibrin (SF), soluble fibrin monomeric complexes (SFMCs), prothrombin fragment 1+2 (PF1+2), D-dimer, and a reduction in levels of physiological blood coagulation inhibitors [4,5,6,7,8]. Understanding and properly evaluating the inhibitory potential of antithrombotic factors is essential, as the anticoagulant component of the hemostasis system serves as a robust antithrombotic shield, which is critical for preventing pathological thrombus formation [9,10,11].

The main physiological anticoagulants that prevent the formation of fibrin thrombus are protein C (PC) and antithrombin III (AT-III). They target the active forms of procoagulant blood clotting factors, with AT-III inhibiting most of them. Conversely, activated PC, in a complex with its cofactor protein S, functions as a potent anticoagulant by selectively neutralizing factors Va and VIIIa, thereby mitigating the potential of the coagulation cascade and excessive thrombin generation. Additionally, it stimulates the fibrinolytic system and exhibits a cytoprotective effect via proteinase-activated receptors (PARs) [12,13,14]. Protein C deficiency can contribute to hemostatic imbalance and serve as a potential trigger for thrombotic complications, as indicated by a reduction in protein S content or activity [15].

Currently, most diagnostic protocols lack the routine assessment of markers for the anticoagulant component within the hemostasis system. However, the diagnostic potential of this anticoagulant pathway, representing the counterbalance to the activation of the hemostasis system, provides valuable insights into the degree of coagulation activation. It can serve as a prognostic indicator for the risk of developing thrombotic complications and is essential for appropriate antithrombotic therapy.

The aim of this study was to assess the alterations in PC levels in pathologies associated with inflammatory processes and the risk of intravascular thrombus formation. A particular focus was placed on the correlation with the severity of the disease course, the intensity of the inflammatory response, and the extent of pathological activation of the blood coagulation system.

## 2. Results

The evaluation of the coagulation component of the hemostasis system included the following tests: determining the fibrinogen concentration, soluble fibrin (SF), soluble fibrin-monomer complexes (SFMCs), and the content of protein C (PC) and antithrombin III (AT-III) in blood plasma.

### 2.1. Systemic Lupus Erythematosus (SLE)

The coagulation system was studied in patients with SLE (n = 192). It was found that the most common coagulation abnormalities in SLE patients included hyperfibrinogenemia (22.9% of patients) and a reduction in PC levels (53.1% of patients), attributed to its consumption during coagulation activation. A decrease in AT-III was observed in 4.7% of patients. Activation of the coagulation process was further confirmed by a significant accumulation of SFMCs in 54.4% of patients (Table 1).

A decrease in PC level was found to be associated with the intensity of the inflammatory process, as determined by the SLEDAI index. Specifically: only 31% of patients with an SLEDAI index < 14, and 74% of patients with an SLEDAI index > 23, showed a significantly reduced level of PC. Additionally, the reduction in PC levels was correlated with the accumulation of SFMCs, a marker of intravascular thrombin generation.

Our studies revealed that SFMCs are present in the blood plasma of only 26% of patients with an SLEDAI index < 14. In contrast, 60% of patients with an SLEDAI index > 23 showed the presence of SFMCs, and in these patients, the SFMC level increased significantly, reaching up to 150 μg/mL. This finding indicates that a decrease in PC level and an elevated concentration of SFMCs reflect increased inflammatory processes and coagulation activation in SLE.

### 2.2. Ischemic Heart Disease (IHD)

The status of the blood coagulation system was studied in 124 patients with ischemic heart disease, including 54 patients with acute myocardial infarction (AMI), 43 with unstable angina, and 27 with stable angina (Table 2).

A comprehensive analysis of the blood coagulation system indicators in stable angina patients revealed no significant abnormalities. Only 1.5% of patients showed the appearance of SFMCs and a decrease in the PC level. In contrast, in patients with unstable angina, 50% exhibited a significantly reduced level of PC, an increased fibrinogen content, and an accumulation of SFMC.

With AMI, an increase in the content of SFMCs was found in 84% of patients, and a decrease in the level of protein C was identified in 74% of patients. Thus, there is a notable difference between the various types of coronary heart disease. Stable angina did not lead to a decrease in protein C levels, whereas in unstable angina and AMI, the protein C content sharply decreased by 60%, indicating a significant pathological activation of the blood coagulation system.

Similar to the case of SLE, the decrease in protein C levels was correlated with an increase in the concentration of SFMC and the severity of the disease.

### 2.3. Abdominal Aortic Aneurysm (AAA)

The blood coagulation system of 20 patients hospitalized for abdominal aortic aneurysm was studied. Table 3 presents the results of the blood plasma analysis from these patients prior to surgery.

We observed a significant elevation in fibrinogen concentration in the blood plasma of patients with AAA (up to 7 g/L, compared to the normal range of 2–3 g/L). Additionally, SF was markedly accumulated in 70% (above 6 µg/mL) of patients (ranging from 13 to 128 μg/mL, with a normal level of 3 μg/mL). In comparison, the PC level was reduced in 45% of patients. These findings highlight the severity of the pathology and underscore the substantial risk of intravascular coagulation in patients before surgical intervention.

A further decrease in PC levels was observed following aortic aneurysm repair (Table 4). Notably, patients with significant blood loss (exceeding 1.5 L) exhibited substantially lower postoperative PC levels (as low as 37%) compared to those with less blood loss (under 1.5 L). This reduction in PC levels correlated with an accumulation of SF, which reached extremely high levels after endovascular repair, with a median of 57 μg/mL and values as high as 199 μg/mL.

Notably, all patients who experienced severe bleeding (greater than 1.5 L) had preoperative PC levels below 84% (n = 7). In contrast, patients with preoperative PC levels within the normal range (n = 13) experienced less bleeding (under 1.5 L), and the increase in SF levels was less pronounced, with a median of 20 μg/mL and a maximum of 106 μg/mL after surgery. These findings underscore the importance of PC level assessment in identifying high-risk patients requiring more meticulous and targeted management to prevent complications.

## 3. Discussion

This study aimed to prove the diagnostic significance of PC concentration monitoring in pathological conditions associated with the risk of intravascular microcoagulation. The prognostic and diagnostic significance of PC was studied in patients with IHD, SLE, and AAA.

In all the studied pathological conditions, there was a correlation between the decrease in PC concentration and the accumulation of SF (or SFMCs)—the verified marker of the danger of intravascular thrombus formation. This indicated that the physiological generation of intravascular active thrombin leads, on the one hand, to the formation of fibrin oligomers and, on the other hand, to the consumption of PC, which performs its anticoagulant function.

Another important finding was the evident reduction in PC levels in patients, which correlated with the severity of the disease.

In individuals with SLE, PC level was closely associated with the severity of the inflammatory autoimmune process. The advanced disease progression correlates with the diverse and sometimes opposing disruptions within the hemostasis system, ultimately contributing to the development of thrombophilia [16,17].

Similarly, the most significant decrease in PC levels was observed in patients with AMI and unstable angina. It is well established that cardiovascular pathology triggers systemic and localized inflammation [18,19,20]. Considering that the progression of such pathologies is closely tied to the inflammatory response, it is unsurprising that no reduction in PC levels was detected in patients with stable angina.

A characteristic complication of AAA is embolization, which involves arterial blockage by a thrombus resulting from disrupted hemostatic balance [21,22,23]. Consequently, the observed decrease in PC levels in patients with AAA was anticipated. Furthermore, we identified a correlation between the extent of PC reduction and the volume of blood loss following aortic aneurysm repair.

Interestingly, the preoperative PC levels were significantly lower in patients who later experienced substantial blood loss following surgery. The volume of blood loss is widely recognized as a consequence of the aneurysm size and the local generation of inflammatory markers [24]. Up to 10% of AAA patients exhibit severe inflammation [25]. Additionally, endovascular repair in AAA naturally triggers an inflammatory response [26]. This supports the conclusion that inflammation likely plays a significant role in the observed decrease in PC levels.

In general, our findings suggest a significant underappreciation of the importance of the anticoagulant system. Commonly performed characterization of the prothrombotic profile of patients suffering from the inflammatory pathologies associated with thrombotic complications is limited to assessing the degree of coagulation system activation. Our results demonstrate that it is essential to assess not only the degree of coagulation system activation but also its balance with the levels of physiological blood clotting inhibitors, particularly PC [27,28].

The specific function of PC lies in balancing coagulation activation by proteolysis of the activated factors Va and VIIIa. This leads to a decrease in the procoagulant potential by suppressing of generation of factor Xa and thrombin. Additionally, PAR-1 cleavage by aPC leads to cytoprotective, anti-inflammatory, and barrier-protective effects. Therefore, the level of PC indicates not only the generation of active thrombin, which can be also indicated by SF accumulation, but the ability of the anticoagulant system to compensate a procoagulant shift in hemostasis. This finding provides strong support for our hypothesis regarding the diagnostic value of PC levels in assessing the blood coagulation system.

**Figure 1:**
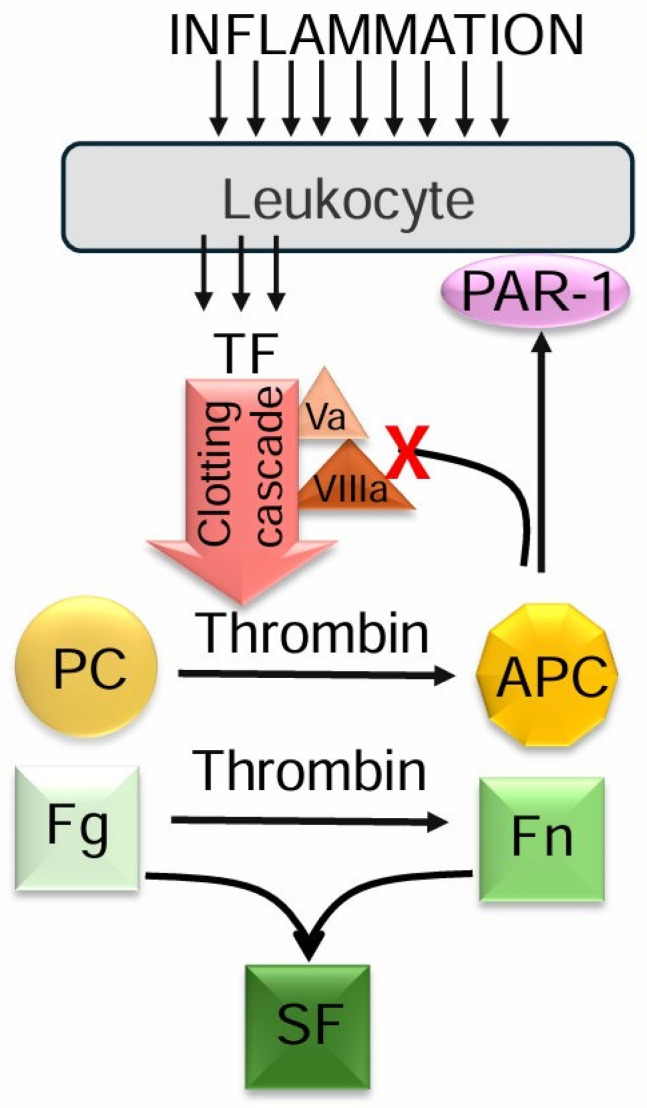
A schematic representation of the relationship between inflammation and hemostasis, highlighting the role of protein C. Inflammation activates leukocytes, leading to the expression of tissue factor (TF), the primary trigger of the clotting cascade, which ultimately generates thrombin. Thrombin, the central enzyme of blood coagulation, converts fibrinogen (Fg) into monomeric fibrin (Fn). In the early stages of this process, monomeric fibrin can be associated with fibrinogen and other fibrin molecules, forming macromolecular complexes of circulating soluble fibrin (SF). Additionally, thrombin initiates anticoagulant mechanisms by activating protein C (APC), which suppresses clotting cascade factors Va and VIIIa and cleaves protease-activated receptor-1 (PAR-1) on immune cells. This process depletes the total protein C levels. Consequently, protein C concentration serves as a potential indicator of clotting propagation, soluble fibrin accumulation, and inflammation. Abbreviations: APC—activated protein C, TF—tissue factor, PAR—protease-activated receptors, Fg—fibrinogen, Fn—monomeric fibrin, SF—soluble fibrin.

The proposed model is briefly summarized in Figure 1.

It is important to evaluate the components of the anticoagulation system because the anticoagulants are depleted faster than the other components of the coagulation system. Under pathological conditions, the formation rate of physiological anticoagulants can significantly lag behind their depletion, underscoring the need for continuous monitoring and timely antithrombotic therapy [29,30].

## 4. Materials and Methods

### 4.1. Materials

Chromogenic substrates S2238 (H-D-Phe-Pip-Arg-pNA), and S2236 (p-Glu-Pro-Arg-pNa) were purchased from BIOPHEN (Neuville-sur-Oise, France); sodium citrate and phosphate-buffered saline (PBS) tablets (pH 7.2, sodium chloride, 0.15 M) were purchased from Sigma-Aldrich (St. Louis, MO, USA). APTT-reagent, control donors’ blood plasma, and test-systems for determining protein C and antithrombin III levels were purchased from Siemens-Biomed (Marburg, Germany). The thrombin-like enzyme was purified from the venom of *Agkistrodon halys halys* according to the method described in [31]. Monomeric fibrin desAB was purified according to the method described in [32].

### 4.2. Patient

#### 4.2.1. Systemic Lupus Erythematosus (SLE)

A total of 192 patients with systemic lupus erythematosus (SLE), aged 15 to 76 years, were included in the study. The cohort primarily comprised individuals from the central and western regions of Ukraine. The diagnosis of SLE was determined using the American College of Rheumatology (ACR) criteria established in 1997. It was formulated following the classification guidelines recommended by the Association of Rheumatologists of Ukraine in 2002. The diagnosis of antiphospholipid syndrome (APS) was made based on established diagnostic criteria. The diagnosis of definite APS was made in patients meeting at least two clinical criteria, combined with high levels of anti-cardiolipin IgG antibodies in serum. Probable APS was identified in cases where either two clinical criteria were met alongside moderately elevated anti-cardiolipin IgG antibody levels or high levels of these antibodies accompanied one clinical criterion. The activity of SLE was assessed using the SLE Disease Activity Index (SLEDAI) [33,34].

#### 4.2.2. Ischemic Heart Disease (IHD)

The study involved 124 patients under the age of 70 diagnosed with large-focal and transmural ischemic heart disease (IHD), without signs of cardiogenic shock. The patients received treatment at the National Scientific Center “M.D. Strazhesko Institute of Cardiology,” National Academy of Sciences of Ukraine, in Kyiv.

#### 4.2.3. Abdominal Aortic Aneurysm (AAA)

A total of 20 male patients with abdominal aortic aneurysm (AAA), with an average age of 62 years, were examined. Among them, 2 patients had suprarenal aneurysm localization, 2 had juxtarenal localization, 2 had ruptured aneurysms, and 17 had infrarenal localization. During surgery, all patients received an infusion of heparin solution at a dose of 0.5–1 mL (2500–5000 IU). Postoperatively, they were prescribed Clexane at a dose of 0.2–0.4 mL for 5 days. To prevent postoperative bleeding, aminocaproic acid and antifibrinolytic drugs were used.

### 4.3. Methods

#### 4.3.1. Blood Plasma Preparation

Platelet-poor blood plasma was prepared from citrated blood by centrifugation at 1200 g during 30 min. Sodium citrate (3.8%) added immediately after collection to the whole blood at 1:9 ratio was used as an anticoagulant.

#### 4.3.2. Fibrinogen Concentration

Fibrinogen concentration in the blood plasma was determined by the modified spectrophotometric method. Blood plasma (0.2 mL) and PBS (1.7 mL) were mixed in a glass tube. Coagulation was initiated by the addition of 0.1 mL of thrombin-like enzyme from the venom of *Agkistrodon halys halys* (1 NIH/mL), which made it possible to avoid fibrin cross-linking. The mixture was incubated for 30 min at 37 °C. The fibrin clot was removed and resolved in the 5 mL of 1.5% acetic acid. The concentration of protein was measured using spectrophotometer POP (Optizen, Daejeon, Republic of Korea) at 280 nm (ε = 1.5) [35].

#### 4.3.3. Activated Partial Thromboplastin Time

Activated partial thromboplastin time (APTT) was performed through the following procedure: 0.1 mL of studied blood plasma was mixed with an equal volume of APTT-reagent and incubated for 3 min at 37 °C. Then, the coagulation was initiated by adding 0.1 mL of 0.025 M solution of CaCl_2_, and the clotting time was monitored. The clotting time was evaluated using a coagulometer CT2410 (Solar-STS, Kharkiv, Ukraine).

#### 4.3.4. Soluble Fibrin Monomeric Complexes (SFMCs)

For the SFMC measurement in the glass tube, 0.25 mL of studied blood plasma sample was mixed with an equal volume of 0.1 M KH_2_PO_4_ buffer pH 7.5. Then, 0.4 mL of 1 M KH_2_PO_4_ buffer pH 7.5 was added to the whole volume after gentle mixing. The samples were incubated for 30 min at ambient temperature. After incubation, the accumulation of saturated SFMCs was estimated semi-quantitatively in the concentration range of 0.007–0.14 mg/mL. For the calibration, we used samples of blood plasma with monomeric fibrin desAB added at final concentrations ranging from 0.007 to 0.14 mg/mL, prepared according to the method described in [36].

#### 4.3.5. Soluble Fibrin (SF)

Soluble fibrin was detected using sandwich ELISA with monoclonal antibodies produced at the Palladin Institute of Biochemistry of NAS of Ukraine. Fibrin-specific monoclonal antibody I-3C was used as a catch-antibody. Biotinilated monoclonal antibody II-4d with epitope in NH_2_-terminal fragment of γ-chain of D-region of fibrinogen molecule was used as a tag-antibody [37]. Optical density was measured at 450–630 nm using multiplate reader RT 2100C (Rayto, Shenzhen, China). Monomeric fibrin desAB was used for the calibration [32].

#### 4.3.6. D-Dimer in Human Blood Plasma

D-dimer was detected using sandwich ELISA as described above for soluble fibrin with modification: DD-specific monoclonal antibody III-3B, with an epitope in the NH_2_-terminal fragment of the Bβ-chain from the D-region of fibrin(ogen), produced at the Palladin Institute of Biochemistry of NAS of Ukraine, was used as the catch-antibody. D-dimer (produced from the human cross-linked fibrin according to the method described in [38]) was used for the calibration.

#### 4.3.7. Protein C Level

The PC level was determined using the PC activator (Siemens, Munich, Germany). The generation of activated PC was measured by chromogenic substrate assay using specific chromogenic substrate S2236 (p-Glu-Pro-Arg-pNa). The analysis was performed in 0.05 M Tris-HCl buffer pH 7.4 at 37 °C. The chromogenic substrate concentration was 0.3 mM. The generation of para-nitroaniline was measured at 405 nm on a microtiter plate reader Multiscan EX (Thermo Fisher Scientific, Waltham, MA, USA). The results were presented as the ratio of the PC level in the blood plasma of the patient to the PC level in the blood plasma of the healthy control. This method made it possible to determine the level of remnant PC that was not depleted and can theoretically be activated.

#### 4.3.8. Statistics

Statistical data analysis was performed using the Wilcoxon–Mann–Whitney (WMW) test and the student’s t-test. All the blood coagulation assays were replicated thrice. The results are presented as 55 means ± standard deviation. Data were considered significant when *p* < 0.05.

## 5. Conclusions

The function of PC in anticoagulation, alongside its role in the anti-inflammatory response, makes it a highly informative diagnostic parameter for assessing both coagulation and inflammatory status in various pathological disorders associated with the risk of intravascular thrombus formation.

## Figures and Tables

**Table 1:** Characteristics of the hemostasis system in patients suffering from systemic lupus erythematosus. The median value and the range (minimum–maximum) are presented. APTT—activated partial thromboplastin time; SFMCs—soluble fibrin monomeric complexes; AT-III—antithrombin III, PC—protein C.

Parameters		Fibrinogen, mg/mL	APTT, s	SFMCs, μg/mL	AT-III, %	PC, %
**Patients with SLE**	SLEDAI ˂ 14 (n = 41)	2.5 (1.2–7.3)	45 (28–67)	7 (0–70)	93 * (71–123)	80 * (42–120)
	SLEDAI 14–23 (n = 108)	2.9 (8.5–0.6)	46 (75–29)	7 (0–150)	97 (76–128)	76 * (45–120)
	SLEDAI > 23 (n = 43)	2.9 (1.2–6.1)	46 (30–110)	35 *^#^ (0–150)	94 (60–118)	72 * (41–110)
**Healthy donors** **(n = 10)**		2.6 (2.2–3.1)	45 (42–48)	2 (1–3)	100 (90–120)	100 (90–110)

*—The data were considered significantly different, as determined by the Mann–Whitney U Test (*p* < 0.05), when compared to healthy donors; ^#^—different significantly according to the Mann–Whitney U Test *p* < 0.05 compared to the SLEDAI group ˂ 14.

**Table 2:** Characteristics of the hemostasis system in patients with ischemic heart disease. The median value and the range (minimum–maximum) are presented. APTT—activated partial thromboplastin time; SFMCs—soluble fibrin monomeric complexes; AT-III—antithrombin III; PC—protein C.

Parameters	Fibrinogen, mg/mL	APTT, s	SFMCs, μg/mL	AT-III, %	PC, %
**Patients with stable angina pectoris** **(n = 27)**	2.9 (1.6–5.4)	50 * (39–99)	0 (0–45)	96 (80–100)	99 (63–100)
**Patients with unstable angina pectoris (n = 43)**	2.7 (1.5–4.1)	60 * (33–120)	25 *^#^ (0–140)	85 * (50–120)	60 *^#^ (45–100)
**Patients with acute myocardial infarction (n = 54)**	3.1 * (2.2–5.5)	42 * (23–71)	45 *^@^ (0–140)	86 (48–120)	63 * (40–110)
**Healthy donors (n = 13)**	2.6 (2.2–3.1)	45 (42–48)	2 (1–3)	100 (90–120)	100 (90–110)

*—Significant according to the Mann–Whitney U Test, *p* < 0.05 compared to healthy donors; ^#^—Significant according to the Mann–Whitney U Test, *p* < 0.05 compared to the stable angina pectoris group; ^@^—Significant according to the Mann–Whitney U Test, *p* < 0.05 compared to the unstable angina pectoris group.

**Table 3:** Characteristics of the hemostasis system in patients with abdominal aortic aneurysm (AAA). The median value and the range (minimum–maximum) are presented. APTT—activated partial thromboplastin time; SF—soluble fibrin; AT-III—antithrombin III; PC—protein C.

Parameters	Fibrinogen, mg/mL	APTT, s	SF, μg/mL	AT-III, %	PC, %
**Patients with AAA** **(n = 20)**	4.0 * (1.8–7.0)	nd	8.4 * (2.0–128.0)	nd	87 * (40–119)
**Healthy donors** **(n = 10)**	2.5 (2.3–3.0)	45 (41–49)	3 (1–9)	100 (90–120)	100 (90–110)

*—Significant according to the Mann–Whitney U Test, *p* < 0.05 compared to healthy donors.

**Table 4:** Characteristics of the hemostasis system in patients with abdominal aortic aneurysm (AAA) after endovascular repair. The median value and the range (minimum–maximum) are presented. APTT—activated partial thromboplastin time; SF—soluble fibrin; AT-III—antithrombin III; PC—protein C.

Parameters	Fibrinogen, mg/mL	APTT, s	SF, μg/mL	AT-III, %	PC, %
**High blood loss** **(n = 7)**	3.4 (1.7–6.2)	nd	57.0 *^#^ (10.3–199.2)	nd	63 *^#^ (37–85)
**High blood loss** **(n = 13)**	3.1 (1.3–9.8)	nd	19.5 * (3.7–106.6)	nd	84 * (70–100)
**Healthy donors** **(n = 10)**	2.5 (2.3–3.0)	45 (41–49)	3 (1–9)	100 (90–120)	100 (90–110)

*—Significant according to the Mann–Whitney U Test, *p* < 0.05 compared to healthy donors; ^#^—Significant according to the Mann–Whitney U Test, *p* < 0.05 compared to the group with low blood loss.

## Data Availability

Upon additional request, the original research row data will be uploaded to the Institute of Biochemistry library Repositorium to make them available to the scientific community.

## Abbreviations

The following abbreviations are used in this manuscript:

SFMCs	soluble fibrin monomeric complexes
SF	soluble fibrin
PF1+2	prothrombin fragment 1+2
SLE	systemic lupus erythematosus
APS	antiphospholipid syndrome
SLEDAI	SLE Disease Activity Index
AMI	acute myocardial infarction
AAA	abdominal aortic aneurysm
APTT	Activated partial thromboplastin time
PC	protein C
AT-III	antithrombin III
PAR	proteinase-activated receptors
IHD	ischemic heart disease
